# On Ether and Chloroform

**Published:** 1855-07

**Authors:** Geo. J. Conner


### ARTICLE IV.

### *On Ether and Chloroform.* By GEO. J. CONNER, D. D. S.

OF all the discoveries of science and art, of all the gifts of genius and labor to man, of all the boons conferred by medicine upon humanity, there is none which, in the extent and the utility of its application, and the amount of pain and suffering it has relieved and prevented, can for a moment compare with the discovery and application of anæsthetic agents. Century after century, from the dawn of time for over five thousand years, there was an amount of suffering and pain inflicted upon humanity, even in the performance of a natural function, and in the very measures which were intended to relieve him from greater ills, which, while it rendered the operative portion of the healing art almost an opprobrium, had come to be regarded as one of the necessary consequences of "that first act of disobedience; that forbidden tree whose mortal taste brought down the primeval curse."

Previous to 1846, the agonizing groans, the thrilling shrieks of mortal agony and dread arose daily from tortured thousands, tortured for their good, under the manipulations of the surgeon, or the instrumental interference of the obstetrician, appealing to the sympathies, and appalling the hearts of relatives and friends, and often causing even the practiced hand and the steeled nerves of the schooled practitioner to shudder for the pangs he had inflicted. Human suffering, as a necessary and unavoidable ill, and in its direst forms, the "cunningest product of Divine creative power" mangled and mutilated, torn and writhing, were daily and hourly presented to the world; while with unflinching heart, keen eye and steady hand, the surgeon was forced for mere humanity's sake to probe to its depths the anguished wound, or still further to mutilate the quivering flesh.

It has been indeed through all time the ardent desire of philanthropists to abolish pain in all its forms and under all its circumstances, and the means of dissociating surgery from one of its most fearful terrors has been for a long time the subject of the day-dreams of the benevolent surgeon of every clime and country. It is true, that as with all other discoveries previous to their full completion, we find inklings of the object gained from numerous sources during the past seventy-five years, from the discovery of hydrogen, in 1766, to that of Sir Humphrey Davy, of nitrous oxyd in 1800; but these were but the mere foreshadowings, or at the best, anticipations of better things. They were not fruit, but were only regarded as the unvalued apples dropping idly and uselessly from the tree of knowledge.

For the present day, and for our own country and countrymen, was reserved the inestimable honor and blessing of robbing pain of its worst terrors, and of conferring a boon upon mankind, which, even in the age of steam and electricity, microscopes and printing press, arrests more universal attention, and excites a deeper interest, than any mere physical fact the world has ever developed.

In the year 1846, Dr. Morton, a dentist of Boston, after experimenting upon himself and several of his patients, prevailed upon Dr. I. C. Warren to use the ether inhalation, and on the 10th October, of the same year, he administered it in the Massachusetts General Hospital, for the extirpation of a tumor in the neck. An incision between two and three inches long was made through the integument and the dissection continued through the cervical fascia; and on the 7th November following, the thigh of a young girl was amputated in the same hospital, the patients, no longer sufferers, being perfectly unconscious of the knife. How different the scenes, and what must have been the anxious fears, mingled with hopeful anticipations, and the proud and gratifying triumph of those who witnessed this great desideratum of surgical art thus obtained: who thus saw an agent so powerful as to abolish life and all its manifestations save circulation and respiration, at their will cease its influence and leave the subject without a consciousness of suffering, and with no ill effects resulting from its action. Such was the firm basis and startling point of etherization, and the benefits which have resulted have only been equalled by the rapidity with which its use has extended over the civilized world. Within six weeks after the publication of a paper upon the subject, by Dr. Henry J. Bigelow, it was tested and found strong advocates among the surgeons of London; in less than three months it was introduced into the Parisian hospitals, and rapidly extended its fame over Europe. In twelve months after its first introduction and use, cases were numbered by thousands, and although cavillers at different points still doubted and sneered, it had established itself as a necessary and universal adjunct to the armament of the surgeon, the physician and the accoucheur.

Hitherto sulphuric ether had always been employed, but in the latter end of 1847, J. Y. Simpson, professor of midwifery in the University College of Edinburg, brought forward chloroform. It is obtained by distilling alcohol with chloride of lime, and containing 2 atoms of carbon, 1 of hydrogen, 3 of chlorine. It is a dense, limpid, colorless liquid, readily evaporating, and has an agreeable, fragrant, fruit like odor, and a saccharine, pleasant and aromatic taste. As an anæsthetic, it possesses, over sulphuric ether, the following advantages according to Prof. Simpson.

1st. A greatly less quantity of chloroform than of ether is needed to produce the desired effect, usually about 120 drops of chloroform, some persons requiring much less.

2d. Its action is much more active and complete, from ten to twenty full inspirations being sufficient to narcotize; much of the time of the surgeon is saved, the preliminary stage of excitement being curtailed, and the patient has not the same degree of tendency to exhileration and talking.

3d. Most of those persons who know from previous experience the sensations produced by ether inhalation, and who have subsequently breathed the chloroform, have strongly declared the inhalation and influence of chloroform to be far more agreeable and pleasant than those of ether.

4th. I believe, that considering the quantity requisite as compared with ether, the use of chloroform will be less expensive.

5th. Its perfume is not unpleasant, but the reverse; and the odor of it does not remain for any length of time obstinately attached to the clothes of the attendant, or exhaling in a disagreeable form from the lungs of the patient, as so generally happens with sulphuric ether.

6th. Being required in much less quantity, it is much more portable and transmissible than sulphuric ether.

7th. No special kind of inhaler or instrument is necessary for its exhibition. A little of the fluid or liquid diffused upon the interior of a hollow shaped sponge or pocket handkerchief, or a piece of linen or paper, and held over the mouth and nostrils, so as to be fully inhaled, generally suffices in a minute or two to produce the desired effect. Hence we learn this difference between these two powerful narcotics, although the effects of the inhalation of ether and chloroform are ultimately the same, the difference so carefully stated by Prof. Simpson should always be borne in mind. For while chloroform is much more powerful than ether—while one ounce of ether is generally used, one drachm of chloroform is the proportional quantity—and while the insensibility from ether takes usually about four or five minutes, that from chloroform occupies only about half the time. Sulphuric ether, contains 1 atom of oxygen, 4 atoms carbon and 5 atoms of hydrogen, and is much more stimulant and irritant to the bronchial membrane than chloroform. It is therefore more apt to produce cough, and its inhalation thus interrupted; beside it is less sedative in its action, and is more likely to produce a primary stage of excitement and active intoxication, and is frequently followed by headache, nausea and vomiting. But it is a less powerful, and therefore less dangerous agent, its effects requiring to be watched closely and continually, but not with the same amount of fear and anxiety as in the administration of chloroform; and in the use of either agent, one great requisite is, to avoid producing asphyxia by not preventing entirely the introduction of atmospheric air, but so to proceed as to mingle its vapor with atmospheric air, which is its proper vehicle for its introduction into the lungs. The ultimate effects to be gained by its use is the relief or prevention of pain, the abrogation of all susceptibility to pain, and of all power of appreciating and perceiving it, and this may be obtained either with or without the entire abolition of consciousness. For, says high authority, total unconsciousness or a full anætherization is desirable, and should always be produced if possible in surgical cases, but in obstetrical cases it is not by any means universally sought or required. The first effect is more or less stimulating, the ears ring, confusion of intellect, restlessness, often a numbness of the limbs; but this stage of excitement is very generally so short with chloroform as to be scarcely appreciated. At times, however, although it is usually short, it becomes exceedingly troublesome, the patient being in a state of intoxication, resembling that from alcohol, loud, violent and obstreperous manifestations, are made, and at times requiring manual restraint. In the administration of the agent, the fingers should be kept constantly on the pulse; indeed this should be done by the person administering it, and every beat should be felt and appreciated while the patient is under its influence; the moment the pulse begins to falter, redoubled watchfulness is required or the agent withdrawn for a while, that atmospheric air may be inhaled. In all cases the pulse from beginning to end should be the guide, regulator and criterion, for while the pulse retains its activity and volume the agent can be used without dread. The same author repeats that the mental faculties and volition become first impaired, insensibility and unconsciousness soon supervene, then follows dilatation of the pupil, turning of the eye upwards, muscular relaxation, and at times stertorous breathing.

Anæsthesia has been divided into two stages. The first is characterized either by the incomplete or partial character of the narcotic, or by its brief duration. The blood appears to be insufficiently impregnated, or the vapor has effected only a portion of the circulating fluid, the influence of which upon the brain is soon counteracted by the arrival in the cerebral vessels of fresh and unadulterated blood. This requires for its production a comparatively small quantity and soon subsides, consciousness returning in from two to three minutes, and therefore although sufficient for the extraction of teeth or for many similar operations of not much importance, does not suffice for ordinary surgical purposes. Neither the pulse nor the pupil is necessarily effected in this stage, the muscular fibre still retains its nervous energy, and is generally somewhat rigid, resisting, in a measure, flexion and extension, and occasionally exhibiting the phenomena of catalepsy, retaining any position given to the body or limb. It is to the first stage that belong those remarkable physiological phenomena which have excited so much surprise in the history of etherization, when the intellect and sensation, cognizance, and memory, will, and action, appear to be and are dissevered from each other; where the patient is conscious of the use of the knife, or of the extraction of teeth, or of expulsive efforts in parturition, but feels no pain, or where it is felt, as evinced by groans or cries, but there is no recollection of it afterwards; or which is more rare, yet the most unpleasant, where pain has been felt and appreciated but with no power to move. These things rarely occur now, when the use of these agents are well understood, but were frequent in their infancy and still at times attract attention. That it has been used in dental operations is true, but that it is attended with more than ordinary danger when thus administered, has been the experience of all who have written upon the subject, and although the history of its use records numerous cases of success in its practice, yet from the following effects of anæsthetics on the human organization we must conclude their administration to be highly dangerous.

Dr. Bond, in his Dental Medicine, has enumerated the following observations, conditions, forbidding the use of anæsthetics.

1. To persons laboring under organic diseases of the heart or great vessels.

2. To persons who are subject to cerebral congestions, or whose brain is in any way unhealthy. Epileptic and hysterical patients are not proper subjects for anæsthesia.

3. To persons disposed to hæmoptysis or to pulmonary congestion and to phthisical patients in all stages of that disorder.

4. To feeble persons, with a weak circulation; to the chlorotic or anæmic.

5. It must be remembered that the nervous system is much more easily and readily impressed by medicinal agents, and especially by narcotics, soon after bleeding; and when the patient has evidently been weakened by recent loss of blood, anæsthetics should be administered with great caution, if at all.

But Dr. J. Y. Simpson, Dr. Flagg, Dr. Channing and numerous other writers on the subject, have mentioned quite a number of successful dental operations, one of which I will relate, was noticed by Dr. Simpson; the subject was a young dentist, who had two teeth extracted, one under the influence of ether, the other under the influence of chloroform. He had an upper molar tooth extracted under the influence of ether. The inhalation was continued for several minutes before he presented the usual appearances of complete etherization; the tooth was extracted, and although he did not feel the least pain, yet was conscious of the operation being performed, and was quite aware when the crash took place. Some days after he required another molar tooth extracted; he inhaled the vapor of chloroform, half a drachm being poured upon a handkerchief for that purpose, and held to his nose and mouth. Insensibility took place in a few moments, but this time he was so completely dead, that he was not in the slightest degree aware of any thing that took place. The subsequent stupefying effects of the chloroform went off more rapidly than those of ether, and he was perfectly well and able again to go to work in the course of a few minutes. The great object to be gained on all operations of the mouth, is the abolition of pain, and the sensibility of the patient to assist in the flow of blood; if complete insensibility is induced, pain is obliterated, but the great danger here is, asphyxia is caused by the flow of blood into the trachea, preventing thereby free respiration, and the use of the adjoining organs.

Again the action of anæsthetics on the nervous system is powerfully depressing, modifying the circulation, and the action of the vessels through which the blood passes. This depressing condition affects first the cerebral lobes, which lose their power, and intellect is impaired; then the cerebellum is affected and the power of regulating locomotion is lost; afterwards the spinal marrow, and sensation and motion are gone; lastly, the medulla oblongata, the motive power of respiration, &c. Now if this be the effect of the narcotic, as stated, on the several organs of power, sensation and life, it does seem highly dangerous to administer it in any but the recumbent position.

To administer it in an upright position would be at once to deprive the patient of the free action of the heart, in sending to the head its due supply of blood necessary to keep up the vital action of the organs there, which being cut off, produces exhaustion, syncope and death. But again, the nature of the anæsthetic state, when fully etherized, very much resembles that of deep alcoholic intoxication: indeed when completely under its influence, the patient is, to all intents and purposes, dead drunk. Ether and chloroform being subtile liquids, with vapors of great tenuity, inhaled into the bronchial tubes, they permeate the lining membrane, enter the blood, and circulating directly through the cavities of the heart to the brain, produce there their specific effects. There seems to be a very strong analogy between the effects of ether and chloroform thus introduced into the system, and alcohol taken into the stomach. Both, in small quantities, produce exhiliration; both, in large quantities, produce the phenomena of dead drunkenness and insensibility to pain. With alcohol, the state continues while the fluid remains in the stomach, and patients have been at once aroused by the use of the stomach pump. In like manner, anæsthesia continues as long as the vapor fills the lungs—respiration pumps the vapor from that receptacle, and gradually aerating the blood, terminates the anæsthetic state. Alcohol is found in the blood by chemical analysis. Ether is equally detected in it by its peculiar odor. The question may then be asked, if alcohol can be administered with little danger, in any quantity and in any position, might not ether or chloroform be given in sufficient quantity and in any position, to induce anæsthesia with as little danger and similar results? In ordinary cases, there is no great solicitude for the safety of a patient who is dead drunk from the effects of alcohol—so experience has shown that ether narcotism is very rarely attended with more danger; indeed, if the analogy between the two be correct, there is every reason to believe the effect the same in both cases, and that of anæsthetics to be entirely innocuous.

The second stage which is always sought for surgical purposes, is characterized and identified by muscular relaxation, continued after the first stage is passed; the arms soon become flexible, and at last fall passive and motionless. The commencement of this state of muscular relaxation may be sufficient for short and trifling surgical operations. A few additional inhalations now impregnate the system, so that revival is deferred for some minutes after inhalation has ceased. The recovery, also, is now, not instantaneous, but gradual, evidences being given, as by attempts to swallow, sighing or moving, which warn of the approaching return of consciousness, and of the necessity, if requisite, of now resuming the inhalation. Protracted beyond this point, stertorous breathing commences, while the pulse begins to beat more slowly, and the pupil frequently, but not invariably, is dilated. Now, the surgical period lies between these two points, the indications of returning consciousness, first noticed, on the one hand, and excessive and dangerous narcotism on the other, which is equally manifested by diminution of the force and frequency of the pulse. The point of danger is the lowering of the pulse; as long as this remains good there is no danger, but if once this falters, danger approaches rapidly and death frequently ensues.

There are some diseases in which it is highly dangerous to use either ether or chloroform. But, beside apoplexy, or a tendency to it, cerebral or pulmonary congestion, diseases of the heart and large arteries, and a strong tendency to syncope, or to asphyxia, there seems to be no decided or positive contra-indications. That there are dangers attending the use of both agents is certain, and that life has been suddenly and unexpectedly extinguished in some cases, is equally true, but while many of these instances were due, undoubtedly, to an improper mode of exhibiting it, the cases have been so few in number, as not by any means to invalidate the claims of anæsthetics. It has been asserted by the exclusive advocates of ether, that all the deaths have been attributed to chloroform, and that ether, in proper hands, is entirely innocuous; while on the other hand, the advocates of chloroform, have been equally sanguine with respect to its harmlessness, and successful results when cautiously administered. From the most reliable sources, statistics go to prove that the most successful results, so far as has been ascertained, have attended both, and will bear a comparison with the best medicines of the present age; for while opium and mercury have victimized their hundreds, ether and chloroform can show as clear a bill of health as any other remedy in materia medica.

The records of St. Bartholomew's Hospital, London, point to the successful administration in over 9000 cases, in not one of which, including the aged, the young, the healthy, the infirm, and the asthmatic, has its employment left a stain on its character. Of those who have died under the immediate operation of anæsthetics, those who have attributed their deaths to these agents, in all cases, seem to have forgotten that long before either of them was used, patients died, and not very rarely either, under the operation, or very soon after, from shock or irritation succeeding the shock, some of these undoubtedly, having labored under some constitutional degeneracy or affection of the heart, which rendered them unable to support the shock of the operation. Now, it is in saving from this shock and its effects, that anæsthetics have done so much positive good, and the fact is found in the results of operations, a larger proportion surviving them under like circumstances, under their use, than before their introduction.

Professor J. Y. Simpson, in an address delivered in 1845, in Edinburg, gave some statistical tables of operations performed, of course without ether or chloroform, from which we gather, that of all the cases operated upon from 35 to 40 per cent., proved fatal.

In most capital operations, singly, the mortality is equally high or higher. Amputations of the thigh, according to Malgaigno, 6 out of every 10 were fatal; of the leg, 5½ in 10; of the arm, 4½ in 10. In the Glasgow Infirmary, from 1795 to 1840, 3½ died in every 10 amputations of the thigh; 5½ in 10 of the leg; 4½ in ten of the arm. In the Edinburg Infirmary, for four years ending July, 1843, of amputations of the thigh, leg and fore-arm, nearly 5 in every 10 were fatal. This was before anæsthetics were used. In connection with this there are other tables of 211 operations under the influence of anæsthetics—in the cases of amputations, they show that the general mortality of cases operated upon, in the ordinary way, has been in the Paris Hospital, by Dr. Burguieres, between January, 1836 and 1841, 332 in 858 or nearly 4 in 10, thus corresponding with Dr. Simpson's tables exactly. Of amputations performed with the aid of the agent he gives 45 cases, of which 12 proved fatal, being an average of 1 in 4, less than 3 in 10. Analyzing a series of amputations of the same kind, of the thigh, leg and arm, in those under the influence of anæsthetics, the deaths have been 2 in 5; in those without, 3 in 5, thus giving a decided advantage to those who have advocated the claims of these agents.

